# Bibliographical Notices

**Published:** 1841-07-01

**Authors:** 


### Periscope;

OR,

### CIRCUMSPECTIVE REVIEW.

"Ore trahit quodcunque potest, atque addit acervo."

Observations on the Management of Madhouses, Part the Second. containing an Account of Susannah Roginson, &c. &c. By *Caleb Crowther*, M.D. Formerly Senior Physician to the West-Riding Pauper Lunatic Asylum. Simpkin and Marshall. London, 1841.

Dr. Crowther must be a perfect bugbear to madhouse keepers. He does not spare them. From the larceny of a pig or a pot of beer up to chaining, torturing, or seduction, he hunts them through all their wickedness. He writes always in a passion. We would not for the world pick a quarrel with Dr. Crowther. His very name is severe.

We cannot go through Dr. Crowther's case or his strictures, and must content ourselves with pointing out one or two of the abuses he denounces, or things he recommends. Among the desideranda in Lunatic Asyla, is a well regulated series and system of *manuscript books containing the history and treatment of the patients.*

Dr. Crowther observes :—"Making reports in writing at every visit adds greatly to the daily labour of the attending physician, on which account it has been too much neglected. The governors of madhouses ought, therefore, to afford every facility to physicians who have taken the trouble to make correct written reports, for the purpose of enabling them to form summaries of their medical practice. The object of these remarks is to show that the West-Riding Visiting Justices have made an order calculated virtually to prevent every physician from making any use of his own reports. This order prevents the books, containing the reports, from being removed from the asylum, and no medical man in actual practice can afford to spend so much time in an asylum as will be required to draw up a correct Report. In order to encourage the young physician to make his Reports uniformly in writing, I beg leave to suggest to him, that independent of the advantage that will acrue to medical science, he will derive personal advantage from such Reports. They will preserve him from unjust attacks from inferior officers, and from incorrect judgment from uninformed Visiting Justices.

Some time after I resigned the situation of Physician to the West-Riding Pauper Lunatic Asylum, I applied to one of the Visiting Justices for a loan of the books containing the Reports of the cases of the patients treated by myself. I received a written order for this purpose, which the Directer refused to comply with. I have since heard, that at a subsequent meeting of the Visiting Justices, an order was made to prevent the books from being removed from the Asylum. The application for this order, I hear, was made by the Director. The reasons given for this application were, that the books were liable to be injured, or lost, or wanted for reference, when allowed to be removed from the Asylum. Had the Visiting Justices deigned to hear both sides of the question, they must have arrived at a very different conclusion. Their order is calculated to frustrate the object for which the books were provided. The purpose which I had in view when I made application for the books, was to make a Report of some cases occurring in my medical practice, during the eight years that I attended the Asylum as one of the physicians. To the most ignorant it must be obvious that no man can be so capable of drawing up such a Report as the individual who treated the patients.

The results of hospital practice may, it is true, be given by another; but no one, except the medical practitioner himself, can furnish the reasons which led to a certain practice, nor the causes which favoured its success, or contributed to its failure. No physician in actual practice can afford to spend so much time in an Asylum as will be necessary to obtain the abstract and details requisite for a medical report, but there are many half-hours, or hours, which at home he may be able to devote to this work. Instead, therefore, of throwing obstacles in the way, it is obviously the duty of the Visiting Justice to promote the correct execution of such a work, by affording every possible facility to the physician."

*Of the Causes which prevent the ready detection of Abuses in Madhouses.*

First and foremost of these, Dr. Crowther justly places irresponsibility to public investigation and public opinion. This particularly applies to the visiting justices of an asylum.

" Fearful of losing power, and jealous of each other, they have never yet formed a sub-committee, to whom intricate and important business should be submitted previous to a general meeting, as well as all casual business occurring in the intervals between the regular meetings.

The consequence is, that the Director may secure to himself almost the entire control of an Asylum. The late Director Ellis told me, that if he wanted to obtain any particular object, he applied to one of the Visiting Justices, and persuaded him to sign a written order for it.

In whatever manner one Visiting Justice may have decided, it is, I believe, contrary to etiquette for the body of them to reverse the decision. In this way, not only in casual emergencies, but in daily occurrences, the Director, by applying not to one of the wisest among his masters, but to one who is fondest of power, to one whose ear he has gained, to one with whom he has lived on terms of convivial intimacy, may obtain whatever he wishes for, and secures for himself an ardent defender, however improper may have been the application. Instead of being impartial judges, although in their general conduct in life honest and honourable men, they act as advocates for their friends, and by law no appeal is allowed to the Quarter Sessions or Assizes, however partial or unjust may have been their decision. Self-election serves to perpetuate the evils which clannism among the Justices, and personal intimacy between the governors and governed, have introduced. The appointment of Visiting Justices by rotation, as in Middlesex, is infinitely preferable to self-election.

To convict a public officer in a madhouse, (who has gained the ear of the Governors), of the most palpable crimes, is as difficult as in an unreformed parliament it used to be to convict a member of bribery. Formerly the Justices decided in cases of dispute between the different officers, without confronting the accuser and the accused.

Many other causes contribute to screen a favoured public officer who has done wrong; besides his being protected and defended by his personal friends among the Justices. The investigation or trial takes place in private, instead of being in open court.

There is no public prosecutor, unconnected with and independent of the Asylum, employed to sift out and bring forward the evidence. No previous arrangement is made calculated to elicit truth. The witnesses, being permitted to remain in the Asylum for days before the investigation takes place, are liable to be tampered with, brought forward, or suppressed, as may best suit the interest of the party to be tried.

The Clerk to the Visiting Justices, the person usually employed, besides being dependent upon and subservient to them, is, perhaps, the most intimate friend of the accused. Admitting him to be one of the most upright and honourable among his class, it is impossible for him, under such circumstances, to exercise an unbiassed judgment. Instead of being of counsel against the accused, he insensibly becomes the advocate of his friend. This he may be safely, because there is no one opposed to him to cross-examine the witnesses.

Should any witness of an unblemished character dare to give evidence, criminating a favoured delinquent, he is liable to be calumniated and abused, brow-beaten, bewildered, and dismissed with loss of character; there being no advocate to support him, or to prevent improper and harassing questions being put to him. Under such circumstances, there may be the semblance of a fair trial, when all the most important part of the evidence has been concealed.

Supposing that the strongest evidence be adduced, of the Rules and Regulations having been violated; or that it be proved that persons have, at the County expense, been boarded and lodged at the Asylum for many months together; or even that presents have been accepted from individuals who have received such favours,—what would be the result?

The affair would be overlooked—would be passed over without notice—if a favoured officer were concerned in it; but if a farmer, gardener, keeper, or domestic servant, were to do such things, he or they would be immediately dismissed with loss of character. Thus discontent and dissatisfaction are generated in the Asylum from a persuasion that the laws are not equally administered. To render an investigation efficient, a regular order or method of examinstion ought to be adopted. The business ought to be commenced by examining the chief officers belonging to the Institution: afterwards let the inferior officers and domestic servants be examined in due order. Being uncertain with regard to the evidence which may be subsequently given, the superior officers will be more cautious with respect to their own; they cannot adjust their own to the case in hand. The greater the guilt, the greater will be the effort to conceal it. Unprincipled minds have much greater fear of being detected, than of committing a crime. Notwithstanding the efforts used by those who have abused their trust in Madhouses, in order to impose secrecy on servants, and to impeach their veracity when they cannot silence them, truth will occasionally ooze out. The attempt to prevent public servants from giving information respecting occurrences and events which they have witnessed, contrary to good morals, and contrary to the rules and regulations adopted in well-regulated hospitals, is itself prima facie evidence of guilt. It is to prevent combinations of this kind, that daily visitors become absolutely necessary, and have been generally appointed in all our best-conducted hospitals."

*Daily Visitors.*—By the idle, the inhumane, and the negligent public officers in madhouses, no set of people are so much detested as daily visitors. On the contrary;—to the diligent, the upright, and benevolent, occupying such situations, they are highly acceptable; they co-operate in preventing neglect, fraud and peculation, and in this manner diminish the labour and responsibility of the Hospital Superintendents;—they contribute to the comfort and cure of the patients, to the protection of good officers and servants, and to the detection of bad ones;—they promote impartiality in the administration of justice, by furnishing correct statements respecting matters in dispute;—they prevent waste and peculation;—they detect licentiousness and crime.

The duties of the Daily Visitor are to watch, observe, and report upon the conduct of all the individuals, whether officers, servants, or patients, attending or inhabiting a public hospital. It forms no part of their duty to give orders or to interfere with the occupation of any one. They report what they see amiss, or what they see worthy of praise, in a book appropriated to that purpose; and when required, they attend the Governors, in order to explain and substantiate their reports. The Daily Visitor is admitted at all hours, from the time when the patient rises, until he goes to bed. To insure cleanliness, he visits every part of the hospital, he examines every nook and corner;—he examines all the provisions in the house, cooked and uncooked, and sees that the patients get the proper quantity allotted to them of good and wholesome food;—he examines, also, the furniture and clothes;—he notices the violations of the rules and regulations;—he notices the neglect, absence, or misconduct of every officer or servant;—he uses his best endeavours to prevent waste and peculation;—he inquires whether any person, who is not a patient or a pauper lunatic, resides in the house.

"If I be asked, how are we to obtain male and female Visitors able and willing to perform such duties, I beg leave to refer to the former part of this work, page 90, for a satisfactory answer. Some of my readers may be startled at the suggestion that Daily Visitors can in any way contribute to the cure of the insane. The paradox, however, is not less true than strange. The deranged generally imagine that they have been confined without just cause, and consider every person hostile to them who opposes their liberation. They fancy that the Physician, the Director, the Matron, and the Nurse have an interest in what they say and do, unconnected with the welfare of the patient. Even in early stages of the disease, it affords relief and comfort to the lunatic to find, that he has a friend and a guardian who will listen to his complaints, and encourage him with the hope of speedy recovery. It often happens that these complaints regarding their food, their restraint, and the conduct of the officers and keepers, are completely groundless. It is impossible to trouble the Visiting Justices with matters of this kind. For this purpose an intermediate set of officers is requisite, i. e. Daily Visitors. If then, when the first dawn of reason, the first lucid interval occurs, the patient discovers that he is frequently visited by a friend, a protector, who has no interest except in promoting his welfare, and no motive for attending the Asylum except for his protection, one great source of mental irritation dies within him, and hope and confidence occupy the place of despair, and he now rapidly recovers.

Daily Visitors are of great use in protecting and encouraging good officers and servants, and in exposing bad ones. Where one person only in a madhouse has the ear of the Visiting Justices, he may tyrannize over and insult all the rest with impunity. It is true that a servant discharged by the Director has the power of appealing to the Visiting Justices, but at present his chance of succeeding against the influence of the Director will be very small indeed, whatever be the merit of the case. With the assistance, however, of the Daily Visitor, the position of the servant is materially changed. The Daily Visitor becomes acquainted with the general character and conduct of every person in the house. If a charge be brought against a servant, at variance with his general conduct, he examines the case narrowly, and will generally succeed in developing its merits. Under such circumstances it will not be very easy to illtreat an honest servant, or to promote and patronize a bad one. Supposing that all the servants should receive orders to tell no tales, which in plain English means that they shall tell no unpleasant truths, where there are no Visitors they know very well that if they disobey or be suspected of disobeying, they will not be discharged perhaps for telling an unpleasant truth, but they will be so harassed and provoked in various ways, that their situation will not be any longer tenable; and this may occur when they have acted very meritoriously in discovering peculation or crime."

*Dr. Crowther's Observations on Haslar Hospital, St. Luke's, Bethlem, and Hanwell.*

Dr. C. visited these in August, 1840.

*Haslar Hospital,* a noble building, worthy of the first maritime power in the world, not being wanted for the sick and wounded in the navy, during the time of peace, has been fitted up in a temporary way for lunatics, both officers and men belonging to the navy. At the time of his visit, this Hospital contained about 150 patients of this description, three of whom were placed under restraint. They do not here possess the same conveniences for restraint which are to be met with in other Madhouses; they have no separate cells for the noisy and violent patients. Manacles and fetters of iron are still in use here, which might advantageously be changed foı leather straps, gloves, and hobbles.

*St. Luke's Hospital* is a very handsome structure, the galleries are lofty and spacious, and the sleeping rooms large and comfortable; the day rooms, from the position of the windows, appeared to me to be dull and dreary. The greatest part of the patients being unoccupied, were sleepy and listless. The position of the building is bad, being in the midst of a noisy population, which is often injurious to the patients. The kitchens and offices are very defective; the airing courts are small in extent, and few in number. Could the site of this Hospital be sold to advantage, it would be desirable to remove this institution to a country situation, where it might have the advantage of larger airing courts, of a garden, and a farm.

*Bethlem Hospital*, as far as regards structure, may be denominated the Palace of Lunatics. Although situated in the midst of a dense population, it has no annoyance from without, like St. Luke's. It has a greater quantity of ground belonging to it, and is completely detached from other buildings. Its airing courts are spacious, and well supplied with convenient seats, and shelter from the sun and rain; but not sufficiently numerous to allow a proper classification of the patients. The galleries are magnificent; they are long, broad, and lofty, and well lighted, and in some degree compensate for the want of more airing courts. In some parts of the building they were removing the only disagreeable object in these galleries, the large iron bars, and substituting for them iron window frames. The large and lofty sleeping rooms contribute, I have no doubt, considerably to the recovery of the patients. The kitchens and offices are ample and convenient. It is impossible for any building, containing the same number of human beings, to be more cleanly than Bethlem Hospital was on the day when I accidentally visited it. I did not visit every cell, but there was no apparent concealment or reserve, great facility was afforded me by the officers for examining every part of it. The water-closets and places where the patients were washed, were particularly clean and inoffensive.

The general aspect of the patients was more healthy than might be expected in so low a situation, and where such a large number are congregated, I was told that the Hospital was generally healthy, and not subject to fever and epidemics. This observation is particularly worthy of notice, because Bethlem Hospital is situated near the river, very low, and in the midst of a dense population. If this situation can be made healthy, any other place in the kingdom, where there is plenty of water, and sufficient fall to admit of proper drainage, may be made so. Indeed, I am convinced that a low situation, with plenty of water and good drainage, is preferable for a prison or hospital to high ground not well supplied with water.

Although a much greater number of patients is occupied in some way or other at Bethlem than at Haslar Hospital or St. Luke's, still there is a striking contrast between the great listlessness of many of the patients at Bethlem, and the happy activity of those at Hanwell. The establishment of keepers and nurses at Bethlem appear to be well selected; they are active and efficient, but inferior to those at Hanwell. The governors and officers at Bethlem Hospital pride themselves upon what has been the greatest bane to that institution, i. e. upon their exemption from public control and visitation. Formerly it was very difficult to obtain permission to visit this place; now there is no obstacle to prevent visitation. From all that I saw in a cursory visit of two hours, I was induced to believe that there is now nothing which requires concealment; nothing of which they need be ashamed. The neglect and disorder in which Bethlem Hospital was found at the time of the Parliamentary investigation, had its origin no doubt in the privacy which had been maintained there; and for the improvements which have taken place, we are indebted to the frequent exposure by the public press of the abuses which have formerly existed there.

Assuming that the site of Bethlem Hospital is healthy, there ought to be a greater number of cures effected there in proportion to the admissions than in any other asylum, excepting those only confined to the reception of recent cases.

The star of Bethlem has long been under a cloud; it has now, however, again become brilliant, and is likely to maintain a conspicuous position among its compeers. At the anniversary festival of the Bridewell and Bethlem Hospitals, held in July, 1840, Sir Peter Laurie, the president, stated, that during the year, 185 had been cured and restored to society. This is highly creditable to the institution, making the number cured amount to 61.66 per cent. This could not have been effected without great attention and exertion on the part of the medical officers. Emulation and competition are now, I trust, likely to become the order of the day throughout all our public metropolitan Madhouses. As far as I am capable of forming a judgment from our statistical details relating to insanity, I do not think it probable that the number of cures under the most favourable circumstances, can ever exceed 70 per cent. It is therefore highly honourable to the officers belonging to the Bethlem Hospital to have made such a near approach to that number. If it be possible to exceed it, it must be in districts where excess is unknown, and civilization much farther advanced than it is at present.

*At St. Luke's*, the number of cures for the year 1839, was, among the men, 46.5, among the women, 69.7, making the average of both 58.1 per cent. This account is taken from the list of patients whose treatment has been completed. The same kind of cases are admitted into both hospitals; whether or not the cures are estimated in the same way the high-minded irresponsible Governors at Bethlem do not deign to tell us; they publish no annual reports. Considering the disadvantages under which St. Luke's labours, the number of cures effected is highly honourable to that Institution. It is remarkable that among the females the amount of cures has been within a fraction of 70 per cent., the number fixed upon by Dr. Crowther as the probable maximum of cures.

*Hanwell.*—" With every thing which I saw at Hanwell I was highly gratified. The situation is elevated, appropriate, and beautiful; the quantity of ground ample, and well laid out; the buildings lofty, spacious, and convenient; the airing courts numerous and cheerful, with suitable conveniences for rest and shelter. The kitchen is the finest thing of the kind which I ever saw. It is very large, lofty, and well lighted, and replete with almost every conceivable convenience. The gardens are extensive, and well cultivated. The workshops are large and well suited for the different purposes for which they have been erected. There are not perhaps so many square feet of air at Hanwell appropriated to each patient as at Bethlem, but the superiority of the situation will no doubt more than compensate for the difference. There is besides, what is of primary importance in all such institutions, an inexhaustible supply of pure water, unaccompanied with stagnant pools or a humid atmosphere.

The keepers and nurses are of a superior order to what I have seen elsewhere. They excel in stature and muscular strength, in personal neatness and dress, in language and manners.

I accompanied Drs. Conolly and Begley in their ordinary morning visit round the Asylum. The patients were very neat and clean, and a great number of them being engaged in work of various kinds, gave an appearance of cheerfulness and animation to the scene, which we shall look for in vain in situations where they are furnished with no employment. They appeared to be less rude and better educated than the pauper lunatics in the north. Their general behaviour towards the Physicians was civil and respectful; they did not seem to stand in awe of them, but received them with evident pleasure and satisfaction as friends coming to administer relief and comfort to them.

Whether the entire abolition of all instruments of restraint will prove advantageous and practicable, I have not had sufficient experience to determine; but of this I am certain, that the public is greatly indebted to Dr. Conolly for making the experiment on a large scale."

Dr. Crowther, though he does seem rather too wroth, is calculated to do a great deal of good.

### An Essay on the Chemical, Botanical, Physical, and Parturient Properties of the Secale Cornutum; with an Engraving. By *T. H. Wardleworth,* Surgeon. Simpkin and Co., 1840.

It is not a little curious that a fungoid excrescence on an ear of corn should have gained, and in a short period of time, a European and indeed a universal reputation for its specific powers over a particular organ in the human body! Yet such is the case. The discovery of its physiological effects belongs to America, and was probably fortuitous. Its chemical composition is as follows:—In one hundred grains of the secale there are 31 of a thick white oil—5½ of osmazome —9 of mucilage—7 of gluten—11 of fungin—3½ of colouring matter—26 of fecula—3 of salts—loss 3½. (Wright's Analysis.)

That such a compound should possess the peculiar property of acting on the muscular fibres of the uterus, and thus expelling the fœtus, is one of the arcana of nature, and may lead us to hope and believe that thousands of potent remedies are yet undiscovered.

"If an infusion of the powdered Ergot be made, and kept covered for a few seconds, on removing the cover should the infusion have assumed a deep pink colour, and the whole of the Ergot having settled to the bottom of the vessel, its powers, as a parturifacient, may then be depended upon. But should the infusion appear milky, and mucilaginous, with portions of the Ergot floating upon the surface, partly soluble, and partly insoluble; if, under such circumstances as these, the Ergot be administered, its effects are seldom if ever produced."

Mr. W. considers the powder as the only efficient preparation of Ergot. It is supposed that the midwives and old women in Germany and France were acquainted with the parturifacient powers of this curious drug, but its employment by the faculty is only recent. The most opposite and contradictory statements and opinions, as usual, prevail among medical men respecting the ergot. One represents it as superseding the use of instruments in all possible cases; another describes it as the "pulvis ad mortem," endangering the life of the mother, and destructive of the vitality of the *child.* A third class of practitioners aver that the ergot is totally inert, and that its effects are purely imaginary! Our author himself, from practical observations, is led to the conclusion that the secale cornutum "has no injurious effects whatever, either upon the mother or the child." Mr. Bower, a practitioner of wide experience in Rochdale, states to our author:—

"Out of 357 cases, of head presentation, in which I have administered the Ergot of Rye, in every stage of parturition, in all states of dilatation of the os uteri, and in many instances of the first child, I never had the slightest reason to regret its exhibition, or believed that it had acted injuriously on the mother or the child, except in two cases. In those cases in which it produced no effect, (being, however, very rare, when properly administered;) or, the effect having subsided without accelerating the delivery, yet I invariably found the labour to progress, or otherwise, as if no interference had been attempted. The children were born alive and vigorous, except where evident signs existed of their having been dead in utero for some time previous to the commencement of the parturient efforts; or where it had been deemed necessary for the safety of the mother, to have recourse to embryotomy, from a contracted or deformed pelvis."

In our author's own practice the ergot has been administered to 1500 patients, without any selection or preference, but "with the most satisfactory results."

"It has been an established practice to administer stimulants in cases of great feebleness and depression; if this be left with the nurse it is often done with indiscretion, if not with injury; but when a considerable degree of faintness is present, with a cold and pallid surface of the body, and the uterine contractions feeble, attended with depression of spirits; then, under these circumstances, stimulants are called for: there has hitherto been no alternative: whatever may be the future evil the present must be overcome, and those stimulants have been habitually given, which act by the fever they create; but Secale Cornutum rouses the system far more certainly than any such stimulant, without raising the pulse; it does not act by a circuitous influence; nor does it induce a morbid arterial action, and thus rouse the torpid powers of the uterus, but its action is direct and specific, and from these circumstances we anticipate its complete adaptation in all natural labours, to the exclusion of stimulants. But we advance a step further, and state that Secale Cornutum is not only the most powerful and safe partus accelerator, but that it is also a powerful dilator of the os uteri."

As an accelerator in natural labour, our author is in the habit of giving 15 grains of the powder in half an ounce of warm water, as soon as the os uteri begins to dilate, and he repeats the dose in a quarter of an hour, if uterine action does not follow. It is seldom necessary, however, to repeat the medicine.

"In most cases a very short period elapses after the first dose before pain is referred to the pubic region, striking from thence to the back; the pains are slight at their commencement, and recur every two or three minutes, gradually increasing in strength and frequency till the os uteri is dilated to the size of a crown piece; the pains after this gradually decline and frequently altogether subside; when this happens the influence of the first dose may be considered as having passed, affording important information; first, that the influence of the medicine is of a limited duration; secondly, that the gradual manner in which the action advances to its full strength and then as gradually abates, is a safeguard in preventing a rupture of the uterus, and in lessening the tendency to hæmorrhage; the slight action at first, prepares the uterus for what follows. The pains having nearly ceased, the patient is in a fit state for a second dose which should be stronger than the first, but not exceeding one drachm, (we have in many cases given two scruples with success,) which renews the action of the uterus, and the child is in a short time expelled."

Mr. W. has followed this practice for twelve years, "and in that period his confidence in the drug has been, without interruption, increasing." Much suffering, he observes, much anxiety, and much time have been saved. But the ergot is applicable to other cases than natural labour. In two cases where premature labour was desirable, he used it with success.

"Both cases were at the seventh month, and to both the draught was administered three successive days, at the end of which time labour commenced, and in ten hours terminated; happily both mothers and children did well. Both the women had a distorted pelvis to an extent which rendered the operation of craniotomy necessary in their previous labours, one having been pregnant three, the other four times: so that the lives of two children have been thus saved to the credit of the means made use of, and with more safety to the patients than any method previously within the power of the medical attendant."

In uterine hæmorrhage, our author considers the ergot as of the first importance, by checking the flow of blood, during or after parturition.

"In presentations of the placenta attended with deficient uterine action, with a considerable degree of depression of the vital powers, I have given the Ergot with the view of inducing that permanent contraction which is so essential to the patient's security."

In descents of the umbilical cord, so low in the vagina as to cause obstruction of the circulation and consequent death of the child, our author has found the ergot of essential service, by insuring speedy delivery.

Numerous cases are detailed in this practical and unpretending little brochure, which ought to be in the hands of every obstetric man in the profession.

A Practical Treatise on Diseases of the Liver and Biliary Passages. By *William Thomson*, M.D., Physician to the Edinburgh Royal Infirmary. 8vo., Maclaghlan and Co. Edinburgh, 1841.

The greater part of this monograph has already appeared in that excellent publication, "The Library of Medicine," edited by Dr. Tweedie, and this circumstance must, of necessity, curtail the range of the present volume, notwithstanding that the "author has been enabled to discuss several topics more fully than was permitted by the limits of that work." The arrangement has also been remodelled, and the value of the book, no doubt, much enhanced by the separate form of publication.

Although there have been many detached papers and essays on hepatic affections during the last 30 years, yet, since the time of Saunders, no complete treatise on the subject has appeared in this country, till the present work was published. The *title* would have been more correct had it included *disorders* of the biliary apparatus, which, in this country at least, are fifty times more numerous than *diseases* of the same. There is not, perhaps, a more widely extended error in pathology, throughout all grades of the profession in Great Britain, than that which respects the supposed prevalence of hepatic *diseases*. Whether Saunders or Currey, or Abernethy, or the host of East India practitioners that have come to reside in this country during the last thirty or forty years, may have originated or sustained this hepato-mania, we do not profess to know; but of this we are certain, that "Liver-Disease" is applied every day to *disorders* of stomach, duodenum, and colon, where even the function of the liver is scarcely implicated, much less its structure. But this is not all. We every day see patients who represent themselves as labouring under "enlargements of the liver," when we find that one of the bellies of the recti muscles has been set down as hypertrophy of the biliary organ.

The work before us is an excellent compilation on the subject of hepatic affections, functional and structural; and, as such, it is infinitely more valuable to practitioners and students than any original essay, however ably executed. But this very circumstance disables us, or any journalist, to offer an analysis of the volume. If we extracted or condensed that which is compiled from others, we should be wasting our time, and if we picked out portions of the original matter we should be doing great injustice to the work itself, by unravelling the thread by which the compiled materials are held together. We cannot do better, therefore, than strongly recommend the work as the best in the English language on the important subject of which it treats. There is one feature, however, in the book which we will just glance at. The able author seems to be afflicted with a complaint not very prevalent in this country, though almost universal on the Continent—the Hydrargyromania. Dr. Thomson has handled this part of the subject with great adroitness—very much in the way which a clever barrister states the case of his client to a jury. The Doctor, however, is more fortunate than the lawyer in this case. The latter is sure to be met by a counter-statement from his brother barrister, that greatly alters the complexion of the case; whereas Dr. Thomson has it all his own way, and certainly he makes the best of it. There can be no doubt that mercury has been abused both in tropical climates and here at home; but we should be extremely sorry to see it discarded from the Pharmacopœia, or an unreasonable prejudice set up against one of the most valuable remedies which we possess.

---

REDSTONE'S GUERNSEY GUIDE; OR, THE STRANGER'S COMPANION FOR THE ISLAND OF GUERNSEY, &c. &c. Duodecimo, pp. 142. Guernsey, 1841.

THIS is a useful little guide to those who visit one of the chief of the Channel Islands; but we can only notice that part of it which relates to climate, in a medical point of view, and which is written by Dr. Hoskins, one of the principal physicians of the place.

Sir James Clark likened the climate of the Channel Islands to that of the adjacent coast of France; but Dr. Hoskins thinks it more correct "to consider the climate of Guernsey as intermediate between that of the adjacent coast of France and the south-western districts of England." It is more mild in Winter than the *former*, and warmer at all seasons than the *latter*, "assimilating more closely to Penzance, and possessing the same peculiarity—warmth during the night." Not a trifling advantage of these islands is that of furnishing *luxuries* at a cheaper rate than the *necessaries* of life can be procured in most other places to which invalids repair.

The temperature is subject to frequent, but not great vicissitudes—the thermometer seldom rising above 80, or falling below 35°—never remaining long stationary, at or below the freezing point. The prevailing winds are westerly; but between the vernal equinox (21st March) and the first week in May, keen easterly winds are frequent, which invalids ought to respect.

"To compensate, however, for these keen though gentle breezes, the usual concomitants of a British spring, the Guernsey summer is delightfully bland and temperate, and its delicious autumn encroaches smilingly into the month of November. So fine is the weather generally for about six weeks, at this season, that it has been proverbially denominated, 'Le petit été de Saint Michel.' From this period until January, the weather is mild but variable, with high, though not cold winds from the westward, accompanied by rain; a combination called in the vernacular, 'Temps de Guernesey.' If there happens to be any cold weather, brief intervals of it now occur until late in February, when bland weather and often warm sunshine prevail, until the middle of March brings a return of the periodical gales."

An unfounded idea prevails that the climate is humid and relaxing; but the annual number of entire wet days are few, and, on the whole, less rain falls in these islands than in England. The rain that does fall is in heavy showers, quickly succeeded by sunshine. The climate is considered by Dr. Hoskins as decidedly healthy, the islands being subject to no other epidemics than those of infantile diseases—and these are generally mild in form, and tractable in treatment. Dyspepsia, especially among the country-people, appears to be the most prominent malady, owing, Dr. H. thinks, to bad diet among the peasantry.

"The efficacy of our bland atmosphere in dry bronchial cough has long been acknowledged; it also proves serviceable in almost all cases of irritation in the air-passages, whether accompanied by increased secretion or not. It is eminently beneficial in 'dry asthma,' not merely palliating the symptoms, but, in young persons especially, ultimately overcoming the predisposition to the complaint. To discuss the merits of the climate in consumptive cases would be idle; change of atmosphere can only avail in the earliest dawn of the malady, and that change should be to a decidedly warm temperature. In the advanced stages the advantages should indeed be great, which induced a patient to forego the comforts of home, increased as they are by present improved methods of regulating temperature, for the questionable benefits resulting from a change even to a more genial climate."

We are much obliged to Dr. Hoskins for his concise but interesting sketch of the climate of the Channel Islands.

The Cyclopædia of Anatomy and Physiology. Edited by *Robert B. Todd*, M.D., F.R.S., &c. Illustrated with numerous engravings.

The present number of this Cyclopædia appears to us a very good one. All the articles are written with care, and contain in their respective spheres an extensive amount of information. The very nature of the subjects precludes us from any circumstantial account of them. But we would select one or two passages for notice.

Animal Luminousness is exhibited in a clear manner by Dr. Coldstream. This property of emitting light is almost confined to invertebrata, chiefly marine. We have accounts from several naturalists of certain *fishes* having been seen to give out light while in their native element, and some have conjectured,—but on insufficient grounds,—that all fishes do so. The turtle and a species of toad inhabiting Surinam have been reported to have the same property; and the eyes of some carnivorous mammals appear to emit flashes of light. But we find this function constantly and distinctly manifested only by certain mollusca, insects, crabs, annelida, acalephæ, and zoophytes. The sharks, more frequently than other fishes, are reported as luminous. The light given out by them is said to proceed from their abdominal surface. When large shoals of fishes are swimming rapidly, flashes of light, broad and deep, are sometimes seen about them and are supposed to be emitted by the fishes themselves. These appear occasionally at very great depths. They have been traced in the British seas to shoals of herrings and the coal-fish; and Dr. M'Culloch enumerates also the pollack, the pilchard, the sardine, the whiting, the mackarel and the gar, as being sometimes accompanied by these lights.

*Characters and Properties of Animal Light.*

It is only in its most obvious qualities that animal light has hitherto been the object of scientific research. In colour and intensity it varies very much at different times in the same animal, and still more in different animals. With regard to *colour* the following varieties occur. In *pholas dactylus* the light is bluish-white; in *lampyris noctiluca* it is greenish with a shade of blue; in *l. italica*, bright blue; in *Elater noctilucus*, brilliant green, with spots of "the most beautiful golden blue;" in *Fulgora pyrrhorynchus*, deep purple and scarlet; in marine animals generally it is white with various shades of blue. Doubtless these differences depend chiefly upon the various colours of the integuments through which the light is seen.

In *lampyris italica*, there are alternate emissions and extinctions of the light, which take place with some degree of regularity and seem to be synchronous with the pulses of the circulating current, visible in the wing-cases of this beetle.*

The fire-fly (*Elater*) shews two kinds of light; one constant, like that of the glow-worm, but more feeble: the other a vivid white light suddenly intermitted. Its illuminating power seems to be greater than that possessed by any other animal; the light emitted from its two thoracic tubercles is so great that the smallest print may be read with it; and in the West Indies, (particularly in St. Domingo, where they are abundant,) the natives use them instead of candles in their houses. They also tie them to their feet and heads in travelling at night to give light to their path through the forest. The intermitting of the light in

---

* A species of *lampyris* lately found in New Holland is said also to shine in rhythmical pulses. Isis, vol. ii. p. 245.

this insect is such as to give an observer the idea of a membranous veil being suddenly drawn over the source of light, and then as suddenly withdrawn.

In a species of cancer seen by Smith in the Gulf of Guinea, the light (which seemed to be emitted by the brain) was of a deep blue colour when the animal was at rest; but when it moved, bright coruscations of silvery light were darted from it in all directions. The light of some centipedes inhabiting the islands of the Pacific is of a beautiful emerald-green colour. It is connected with a mucous matter covering the animal, which may be rubbed off by the fingers, and communicates to them a smell not unlike that of muriatic acid.

Sometimes the light proceeding from the sea is so white and dull as to give the effect of a sea of milk. This is frequently seen in the Gulf of Guinea, and seems to be caused sometimes by the presence of numerous *Salpæ* and *Scyllari*, at other times by the admixture of the *debris* of fishes and other marine animals recently dead.

An extraordinary series of phenomena connected with a particular display of the luminousness of the sea, is reported by Mr. Henderson as having occurred in the Atlantic, (lat. 2° long. 21° 20' W.) on the 5th March, 1821. About 9 p.m. the sea appeared unusually luminous. Every person who kept his eye fixed upon it for but a short time was immediately affected with giddiness, headache, pain in the eyeballs, and slight sickness. Although these symptoms varied in intensity amongst the spectators, yet there was not one on board who did not feel some degree of them; and all imputed them to the effect of the light proceeding from the surface of the ocean. Mr. Henderson remarks: "For my own part, the headache, &c. which followed immediately my looking at the water, was particularly severe, nor did it go off until morning. The effects I experienced were like those produced by smoking too much tobacco."

There have been recorded some accounts of very intense light produced over a great extent of the ocean's surface by luminous animals, but it does not appear that any other voyagers have experienced physical effects from the light such as are described by Mr. Henderson. The great intensity with which it is occasionally produced by marine animals, however, is well illustrated by the descriptions that are given of the moral emotions with which it inspires the beholders. Witness, for instance, Mr. Bonnycastle's description of a scene which he met with in the Gulf of St. Lawrence, (7th Sept. 1826.) While it was very dark, a brilliant light, like that of the Aurora, was seen to shoot suddenly from the sea, in a particular quarter. It spread thence over the whole surface of the water between the two shores of the Gulf: and shortly there was presented "one blazing sheet of *awful* and most brilliant light." "Long tortuous lines of light showed many large fishes darting about as if in consternation at the scene." The light was sufficient to enable one to see the most minute objects on the ship's deck. On drawing up a bucketful of the water, and stirring it with the hand, it presented "one mass of light, not in sparkles as usual, but in actual coruscations."

Messrs. Quoy and Gaimard state that in handling luminous marine animals while alive, they have always been sensible of an odour proceeding from them similar to that which is perceived around a highly charged electrical apparatus.

The only observation with which we are acquainted that seems to indicate the evolution of heat in connexion with the light of animals, is that reported by Macartney, who states that he found the thermometer raised by two or three degrees when placed in contact with a group of living glow-worms shining, or even with their light-giving sacs cut off. The repetition of this experiment, however, has not produced the same result in the hands of others: they saw no rise of the thermometer.

By far the greater number of luminous animals with which we are acquainted are natives of warm climates; but those inhabiting the ocean are seen in almost all latitudes, even in the coldest; although in these they are not so numerous, and give less light. No aërial insects give out light, in ordinary circumstances, excepting at a temperature of about 50° Fahr. and upwards; and the higher the natural temperature, the brighter is the light emitted.

Marine luminous animals very readily emit their light on being struck by any moving body; so that one of the most commonly observed phenomena connected with this subject is the sparkling of the minute *medusæ* and other animals, swimming on the surface of the sea, when they are dashed against the sides of a ship, struck by an oar, or tossed on the foamy crests of the waves; and this even while no other light is seen excepting just at the points where the water is agitated.

When any loud noise is made near a luminous insect while shining, it frequently ceases to give out its light.

With regard to insects, we have many concurrent testimonies to the fact that more light is emitted during the season of procreation by most of the species than at other times.

That the luminous function is in many animals directly under the control of their will, seems to be proved by the fact, that while under any sudden irritation calculated to alarm them, they, at first, emit light strongly, yet on the frequent repetition or continuance of the same kind of irritation, they extinguish their light, and cannot be excited to shew it again for a considerable time.

*Effects of Extraneous Circumstances.*—Light giving animals being removed from their natural situations, and subjected to artificial processes and agents, are found to have their luminousness affected by being exposed to, 1. the effects of accumulated electricity and electrical currents; 2. immersion in various fluid and gaseous media; 3. pressure of their bodies; 4. removal of their luminous organs, and mutilation of these and of other organs; 5. exposure to various degrees of heat and moisture; 6. immersion *in vacuo*; 7. removal from all foreign sources of light.

The luminous organs may be cut out from the bodies of glow-worms and fire-flies without the peculiar property of the organs being immediately destroyed. The emission of light can for some time be re-excited by slight mechanical irritations; as by touching the organs with the point of a pin. Those of the glow-worm have been seen to shine for two or three days after excision, when slightly moistened with water, heated or electrified. In experimenting on the same insect, Todd found that the light was extinguished within six minutes after the head was cut off; as also when the luminous rings were cut into, but was renewable by the application of heat. Sheppard removed the luminous matter from a glow-worm; the wounds healed within two days, and the body became again filled with new light-giving substance.

If luminous insects be confined in a dark place, they shine little in the early part of the day, but long before night they begin to do so; although generally, in their native situations, they do not emit light until the twilight. If the confinement in a dark place be protracted, they do not shine so brightly as after having seen the sun during the day.

*Anatomy of Light-giving Organs.*—The researches on this head are imperfect. The following are the results obtained by Macartney and Spix from their dissections of the glow-worm, the fire-fly, and the lantern-fly.

In the glow-worm, there is spread over the internal surface of the segments of the abdomen a yellowish substance of the consistence of paste, which is thickest in the middle of each segment, and terminates near each margin by a wavy outline. It is of a closer texture than the fatty matter, but otherwise resembles it. Besides this substance, the last segment is furnished internally, just beneath the most transparent part of its integument, with two small round bodies, lodged in depressions, which contain yellow matter of more close and homogeneous texture.

Müller and Murray describe these round bodies as "two small ovate sacs, composed of thready membranes, and filled with a soft yellow pasty matter." Under the microscope, they appeared to Macaire to be composed of numerous branching filaments, with minute granules adhering to them. It is from points of the surface corresponding to the situation of these round bodies that the light is most constantly and most brightly emitted. When dry, these luminous organs have somewhat of the appearance of gum. The dried matter is translucent and yellowish, becomes darker on being kept, and appears to be granular in its structure. Its specific gravity is a little greater than that of water.

In the fire-fly, the internal concavities of the yellow spots of the corselet, whence the light proceeds, are filled with a soft yellow substance, oval in shape, and of very uniform consistence and density. This, under the microscope, appears to be formed of a large number of very minute parts or lobules, closely pressed together. Around these oval bodies, the fatty matter of the corselet is arranged in a radiated manner. Spix describes the same organs as "yellowish glandular masses, into which many branches of the trachea enter." In *elater ignitus* the masses of luminous substance are extremely irregular in their figure; they are situated close to the posterior angles of the corselet, and are more loose in their structure than the same parts in *elator noctilucus.*

The luminous proboscis or snout of the *fulgora* is hollow, and has a free communication with the external air by a narrow slit situated near the base of the organ. Its cavity is lined with a fine membrane, between which and the outer translucent corneous crust, there is interposed a soft tissue of a pale reddish colour, arranged in lines longitudinally, which is supposed to be the seat of luminousness in this insect.

*Theories of Animal Luminousness.*—Out of seven, the following three appear the best.

5. That a fluid containing phosphorus is secreted by the luminous organs, and shines on its being exposed to the oxygen of the air introduced by respiration. (Darwin, H. Davy, Heinrich, Treviranus, and Burmeister.)

6. That the luminous organs concentrate and modify the nervous influence so as to form it into light; so that, according to this theory, animal luminousness is an effect *solely* of *vital* power. (Macartney and Todd.)

7. Tiedemann thus expresses his opinion: "Animal luminousness would seem to depend on a matter, the product of the changes of composition accompanying life, and, to all appearance, secreted from the mass of humours by particular organs. This liquid probably contains phosphorus or an analogous combustible substance, which combines with the oxygen of the air or of aërated water at a medium temperature, and thus produces the disengagement of light. The preparation and secretion of this substance are acts of life, which change, augment, or decrease by the influence of external stimulants, whose action on the animals modifies their manifestations of life. But the phosphorescence itself depends on the composition of the secreted matter and cannot be regarded as a vital act; because, on certain occasions, it continues for whole days and even after the death of the animal."

Our author leans to that of Macartney and Todd, but is obliged to prop himself on the chemical too.

*Uses of Animal Luminousness.*—We know nothing certainly with regard to the uses of the light-giving function; but as almost all observers have remarked that male insects seem to be attracted towards their mates by the brilliancy of the light emitted by the latter, it has been generally supposed that the luminousness is subservient to the generative function. Although it may be so to a certain extent, it is obviously not essentially connected with it, even in the glow-worm; for the light endures long after the season of love is past. Some have conjectured that the light may sometimes be the means of preserving its possessors from the destructive attacks of enemies. Thus Sheppard observed a large beetle running round a shining *scolopendra*, as if wishing to attack it, but seeming to be scared by the light. We may imagine, also, that the light enables it possessors to see surrounding objects at night, and so to thread their way in safety through the darkest places.

Considering that, in the ocean, there is absolute darkness at the depth of 800 or 1000 feet, at least that, at such depths, the light of the sun ceases to be transmitted, Maculloch has suggested that, in marine animals, their luminousness may be "a substitute for the light of the sun," and may be the means of enabling them to discover one another, as well as their prey. He remarks, "It seems to be particularly brilliant in those inferior animals which, from their astonishing powers of reproduction, and from a state of feeling apparently little superior to that of vegetables, appear to have been in a great measure created for the supply and food of the more perfect kinds."

Lymphatic and Lacteal System is well described by Mr. Lane. We are induced to quote a passage embodying the anatomical views of this gentleman.

"It has appeared to me in the first place, that anatomists who have especially devoted their time to this interesting subject of late years, have not yet fairly freed themselves from the influence of the Hunterian views with respect to the part performed by the lymphatic vessels, as well as by the arterial capillaries, in effecting the growth and habitual nutrition of the structures. To support the Hunterian theory the lymphatic was required to be present with every molecule of the organization, there with open mouth (for imbibition in the living body was not admitted as possible) to remove the old material in order to make room for the new, which was supposed to be deposited by the open mouths of capillary arteries. Now, although physiologists no longer admit that the arteries any where terminate by open mouths, but consider all nutrition to take place by the transudation of the liquor sanguinis through the delicate tunics of the capillary blood-vessels, and although venous absorption, as well as lymphatic, is acknowledged to take place, consequently that the ubiquity of the lymphatic ceases to be a matter of necessity, still it appears to me that physiologists have not yet shaken off the old impression, that every particle of the organization must have its lymphatic vessel, and I cannot help thinking that the continuance of this impression is misleading us in our notions of the arrangement of the system.

There are also some additional anatomical considerations which have had their weight in leading me to the opinion that the lymphatic system is less extensive than is generally supposed. It is not, I believe, known to anatomists that the lymphatic vessels admit readily of dissection in their uninjected state; these vessels do not easily give way under traction, and by using the forceps to hold them, and a blunt but pointed instrument to detach them from the surrounding cellular membrane, to which they are but loosely attached, they may be dissected with equal facility as the cutaneous nerves, for which they are not unfrequently mistaken by the young dissector. I have in this way several times dissected the lymphatics of the upper extremity, from the glands in the axilla to the fingers, and in the lower, from the inguinal glands to the toes. In proceeding thus to trace these vessels, scarcely a single lateral branch can be detected in the leg and thigh, by which the supposed universal network of the surface of the skin could have been connected with the rest of the system. When the subcutaneous lymphatic vessels are injected with quicksilver, every anatomist must have remarked the absence of lateral branches; this has always been accounted for by supposing a valve at the termination of each lateral branch into the larger longitudinal vessels; but in dissecting these vessels in their uninjected state, the lateral branches if present ought to be met with, which is not the case. I am fully aware that Haase, and other investigators, have succeeded in getting the injection to pass in a retrograde direction from the subcutaneous lymphatics of the lower extremity into a net-work of vessels of small extent situated close to the surface of the skin: this has occurred to myself on two occasions, in the skin over the tibia, and in the inguinal region, but in both these instances it was in a portion of skin presenting a cicatrix; the net-work was circumscribed, and left the impression on my mind of an abnormal rather than of a normal condition of these vessels. The entire profession have adopted the notion that the process of ulceration is effected by the lymphatic vessels, consequently that, as every structure may ulcerate, so it must have its lymphatic vessel. But I may be permitted to ask pathologists to consider, whether they are not still influenced by the Hunterian theory, viz. that the countless open mouths of the lymphatics (which modern anatomists do not allow them to possess) effect the removal of the textures disappearing by ulceration, rather than by the few facts and observations bearing upon this important question. I would ask whether the occasional instances, of inflamed lymphatics containing pus, being found leading from an ulcerated surface, are sufficient to establish the opinion, that the whole process is effected by this set of vessels: or whether the occurrence is not more satisfactorily accounted for, by the supposition that the ulcerative process has implicated a lymphatic vessel, and that the pus has entered the vessel by an opening thus effected in its paries, or that the pus has been formed in the lymphatic itself, as the result of inflammation affecting its interior: more particularly when it is borne in mind, that the pus globule is much too large to have entered these vessels by imbibition, and that open mouths are denied to them. The parts of the body in which I have seen pus in the lymphatics, have been on the surface of the lung, on the mucous membrane of the intestines, on the penis when ulcers had occurred in these organs, also in the subcutaneous lymphatics after suppuration and sloughing of the cellular tissue,—situations in which every anatomist has seen lymphatics, and where the ulcerative or sloughing processes might readily have effected an opening into them."

We again and for the twentieth time cordially recommend this work to the profession.

---

### Comparison of the Sickness, Mortality, and prevailing Diseases among Seamen and Soldiers, as shewn by Naval and Military Statistical Reports. By Major *A. M. Tulloch,* F.S.S. Read before the Statistical Society, 15th Feb. 1841.

The parallel between the two Services could only be extended over the seven years that preceded 1837. The first table in the paper gives the following result, in respect to troops and seamen in the Mediterranean for 1830 to 1837. The mean strength of the naval force was 55,709—of which 72,671 came under medical treatment, deaths 617—invalided 1433. The annual ratio of mortality was 11 $\frac{1}{10}$ per one thousand men. Let us see the military results. The mean strength was 62,300—the number treated for sickness being 67,779—and the deaths 1,270—or 20 $\frac{1}{10}$ per thousand men!!

The striking difference in the ratio of mortality in the two services is attempted to be explained, in part, by the talented author—such as sailors being more subject to slight hurts than soldiers, &c. but still, there must be other and more potent causes in operation than those stated by the Major. The following passage is more to the point.

" To these circumstances, tending to lower the mortality on ship-board, may be added the sanatory influence of the sea-air on those chronic affections of the liver and bowels, from which persons are apt to suffer in warm climates, and which will be adverted to more particularly in the following comparison of the various diseases to which the naval and military forces stationed in this Command have respectively been subject during the period under review."

We may now glance at the comparative prevalence of particular diseases. Thus diseases of the lungs amounted to 13,514 out of 55,709 seamen, of which died 177. The pulmonic diseases in the army amounted to 8,955, in 62,300 men, of whom 405 died—or rather more than double the number in proportion. As a set off, however, we must recollect that there were more lung affections, as compared with the whole force, in the navy than in the army, and the probability is, that they were more slight in their nature, and consequently less fatal in the result.

The statistics of phthisis in the two services are somewhat remarkable. Thus in aggregate naval force of 55,709, there were 285 cases of consumption, of which 105 died. In the army aggregate of 62,300, there were 417 cases of phthisis, of which 272 died—or about two-thirds. In the navy, then, we find that nearly one-half of the cases of consumption recover! A contemplation of this statement plainly tells us that, after all, we must take or make our calculations with considerable caution. Suppose we were to take either the naval or military reports, or both, for our guides in private or in public practice, and were to predict that one-half or one-third of our consumptive patients would recover, what would become of our diagnostic or prognostic reputation at the end of 20 years? It is manifestly evident that the basis of the statistical report was erroneous—and that not one in ten—no, nor one in 30 of the cases were phthisis in reality. If, in this case, then, we detect a most decided error in the basis of the statistical record, are we to fear that similar errors are necessarily incorporated with the other items, and that statistics are not to be depended on? It must be remembered that, in no disease whatever, are there more errors of diagnoses committed, both in public and private—among all classes and ranks of practitioners, than in phthisis. Hence we find quacks and regulars advertise their nostrums, and publish their false doctrines respecting "consumption curable." We must not therefore undervalue statistics, because they tell us, in figures, that the naval doctor cures one-half, and the military one-third of their cases of phthisis. Major T. thinks that the circumstance of invaliding has tended to increase the error.

There seems, however, little doubt that either the sea air or the excitement produced by the voyage, do sometimes operate very materially in alleviating the symptoms of this disease. Many soldiers sent home from Malta, with the apparent symptoms of confirmed phthisis, have arrived in this country in renovated health, and speedily returned to their duty; and so marked has been the improvement in several instances, that within the last year increased facilities have, at the special request of the medical officers, been afforded for sending home invalids of this class. Thus while the faculty in this country are sending their consumptive patients to Malta, the medical officers in that island are sending soldiers labouring under the same disease to England; and as benefit is supposed to be derived from the change in both cases, it seems much more likely to arise from the influence of the voyage than mere change of residence, especially as the proportion of deaths among those labouring under consumption is remarkably low on ship-board."

### Diseases of the Liver.

In the aggregate naval force already mentioned there are 403 attacks of inflammation of the liver, of whom 12 died. In the army (62,300) there were 722

attacks of hepatitis, and 29 deaths. The ratio of mortality, as regards the aggregate strength, was just double in the army, as compared with the navy.

### DISEASES OF THE STOMACH AND BOWELS.

The number attacked by inflammation, dysentery, indigestion, diarrhœa, cholera, and other affections of this class, amounted to 8,649, in the naval aggregate of 55,709—and 52 died. In the military force (62,300) there were 11,737, and 157 deaths. Thus the ratio of fatality was, in the army 2½ per thousand men—whereas it did not amount to quite one in the thousand seamen. This is very remarkable.

Cholera occurred in 96 cases out of the aggregate naval force, with 22 deaths. In the army there were 459 cases, and 131 deaths. Here again the ratio of mortality was greater in the army than in the navy.

### DISEASES OF THE BRAIN.

This class comprehends a number of genera and species, and shewed, in the navy (55,709) 958 cases, with 52 deaths—in the army (62,300) there were only 656, with 67 deaths. Here the proportion of attacks is greatest in the navy, and the mortality least.

In the whole of the naval force during seven years, there were only two cases of suicide—in the army there were 20, independent of several found drowned.

In dropsy (subcutaneous, abdominal, and thoracic) the mortality was just double in the army, as compared with the navy.

The following table shews that there are five kinds of disease (few of which are fatal) which are much more prevalent in the navy than in the army.

| | NAVAL FORCE. Out of an Aggregate Strength of 55,709. | | MILITARY FORCE. Out of an Aggregate Strength of 62,300. | |
|---|---|---|---|---|
| | Attacked. | Died. | Attacked. | Died. |
| Rheumatism .. .. .. | 3,560 | 8 | 2,740 | 4 |
| Syphilis .. .. .. .. .. | 2,771 | .. | 1,522 | 4 |
| Gonorrhœa .. .. .. .. | 1,451 | .. | 2,254 | .. |
| Ulcers .. .. .. .. .. | 3,969 | 6 | 3,508 | .. |
| Erysipelas .. .. .. .. | 531 | 15 | 137 | 4 |

Thus rheumatism is much more frequent, in the naval than in the military service, and well it may be, when we consider the exposure of the seaman to night air and wet weather, while keeping watch and watch every night, while the soldier only mounts guard once in three or four nights, and is generally sheltered from the storm. It is not so easy to account for the greater frequency, and also the greater fatality of erysipelas on board ship, as compared with the same disease among soldiers on shore. Probably the frequency of exposure to wet, cold, and night air, when going aloft or keeping watch, together with the paucity of vegetables and fresh meat, may predispose the sailor to erysipelas more than the soldier. We return Major Tulloch our best thanks for this interesting paper.

An Appeal to Parliament, the Medical Profession, and the Public, on the Present State of Dental Surgery. By *George Waite*, Esq. Surgeon Dentist, M.R.C.S. &c. London: Highley, 1841.

Mr. Waite, who is an accomplished dentist, places the unprotected state of dental surgery, and the impositions on the public that it leads to, in a striking light. The following anecdotes are piquant.

"It will not be inappropriate here to quote a ludicrous scene which occurred a few days since at one of our public offices, shewing the present degraded state of this branch of the medical profession.

'*Magistrate.*—Then after he made three sets of teeth, which were of no use, he persuaded you to go to Mr. ——, whose pupil he said he was?

*Complainant.*—He did.

*Mag.*—Did you go?

*Com.*—Yes.

*Mag.*—What did he say?

*Com.*—That for a hundred guineas more, he would make me a set of teeth with which I could eat beefsteaks, and which would last me my life. *(Loud Laughter.)*

*Mag.*—Did you pay him the money?

*Com.*—Yes.

*Mag.*—Have you a receipt?

*Com.*—No. I asked for one, but he would not give it me, and I have called on him without being able to see him.'

In another instance I was requested by my friend Dr. Scott, to give advice where an elderly and helpless lady had been defrauded of an enormous sum for two most clumsy-looking bits of gold, which it was pretended were worked up at Paris, by some extraordinary workmen, which were to be found nowhere else in the world.

Not long since, I was informed that a gentleman received an account of £170. for work done for his family, which was not worth £10. and which enormous sum was compromised for £100.

A lady, caught by the advertisement of a man calling himself the first operator of the day, and other high-sounding titles, stating that no one in the world but himself knew anything of the art, was compelled to seek redress in a court of law: the verdict was against her, but in the trial it came out that a model was made of her mouth before the teeth were extracted, therefore, how could the work answer. If any dentist of respectability had been subpœned on this trial, the verdict would have been different. We find governesses and servants who have saved money are too often duped by these men, and in many instances they are left wholly destitute.

I refer only to cases which have come within my own knowledge.

The system is not confined to London. Near Oxford, a dentist persuaded a poor groom, who had just received £8. for wages, that unless his teeth were cleaned and some extracted, he would lose his life. He sent him home *minus* the £8. His master is an intimate friend of mine."

Mr. Waite recommends that the legislature demand evidence of qualification, before a licence to practice dental surgery is given. He advises the following system of education.

"But perhaps I might subjoin a view of the system of education which I should recommend a dentist to pursue. Mathematics and mechanical philosophy should form the groundwork of his education. I would urge that it ought to be imperatively demanded by the Medical Reform Bill that he pursue a system of work for three years, at least, to gain a steadiness of hand and thorough knowledge of instruments.

In proof of the necessity for this qualification I may urge a case or two, (one occurred not long since,) where an instrument having slipped, lacerated the soft palate, broke the small bones contiguous to it, and stopped against the base of the skull. This occurred to the daughter of an exalted personage; both however are now no more.

In another instance, a sharp instrument went through the cheek at once, and often instruments have slipped down the side of the lower jaw into the glands under it. Steadiness of hand, which work alone can give, should be an essential consideration. I would have every dentist well versed in chemistry, have attended three courses of lectures on anatomy, physiology, and surgery, and also have pursued hospital practice.

This should qualify him for examination, his licence to practice, or his diploma, should be given to him on his qualifications being proved, and his pupils afterwards always apprenticed to and examined by a board of officers.

There might exist a reserved clause, that all dentists being established at this time, should be examined as to their qualifications, and their age noticed; and wherever gross abuse has existed and can be proved, the licence should be refused."

---

A FEW HINTS ADDRESSED TO MEDICAL STUDENTS ABOUT TO VISIT THE PARISIAN HOSPITALS. By *A Physician.* London: John Churchill, Princes-street, 1841.

THESE hints are mostly worth having. We shall take one or two of them. And first a hint on *lodgings* and *eating*.

It is a matter of experience known to all the medical men of Paris, that a large proportion of the medical students are attacked with fever within a very short period of commencing their studies. Now, though this may be accounted for in many ways, and may arise from various causes, yet it is generally allowed that the situation selected for their lodgings is of itself sufficient to be an exciting cause of disease. A more unhealthy situation than some of those small and narrow streets and alleys that abound near the Ecole de Médecine cannot be conceived. In these parts the medical students chiefly reside, for the sake of convenience and economy: here they congregate together in the miserable *maisons garnies*, living, as they occasionally do, two or three in one apartment. The cheap restaurants or eating-houses that also here abound, only add to the general wretchedness of the scene, wherein, under the semblance of comfort and economy, the student is offered his *dejeuner à la fourchette* for sixteen sous, and his dinner *de quatre plats* for twenty-two sous. To many this may appear of little importance, and to be little connected with the subject under consideration; but reason and experience will draw a different conclusion. Is it possible that the body accustomed to nutritious and healthy food, to all the comforts of English living, to fresh and wholesome atmosphere, can meet with so sudden and so great a change as this, and not be affected by it? What quarter then of Paris is to be selected by the student? If the faubourg St. Germain side of the Seine must be chosen, from its contiguity to the hospitals, the rue de Seine St. Germain, or the rue de Sts. Pères, affords a far more healthy and open situation than the localities previously mentioned. True it is, that apartments may be somewhat more expensive, yet surely it is far preferable to sacrifice a small sum of money for the sake of health than to run the risk of becoming the victim of low typhoid fever. The Tuileries side of the water is colonized to a great extent by the English, and is certainly preferable to the faubourg St. Germain. Here lodgings are to be had tolerably cheap, but depend much on situation. In the faubourg du Roule, the continuation of the faubourg St. Honoré, lodging may be procured at a moderate price, and the situation is very healthy, but it is some distance from Hôtel Dieu, La Charité, La Pitié, &c. It is, however, contiguous to the Hôpital Beaujon, to which M. Louis has lately been appointed, during the alterations that are taking place at Hôtel Dieu. Again, with regard to meals: one wholesome dish in a respectable restaurant's is far better than four offered for the same money in many of the cheap eating-houses. For two francs a good dinner may be procured, and a déjeuner or breakfast for thirty sous, in many a clean and wholesome restaurant's. The great difference that exists between French and English living must, more or less, affect every constitution, however strong it may be. It is scarcely possible to conceive of two greater extremes. In Paris there are a set of small hotels, which profess to give English dinners. These are generally as dear as they are bad; but one or two are to be found in the neighbourhood of the Boulevards des Italiens, where, at times, a tolerable English dinner is to be met with: in these, as must of necessity happen in places of like description, the company is not of the most select order; but if this can be dispensed with, it is certainly worth while of those who are suffering from the dyspepsia of Parisian cookery to try for a time the change.

*Walking the Hospitals* should not be entered upon for the first three weeks after the arrival of the student in Paris. The atmosphere of Paris is very peculiar, and this is exemplified in the numerous patients that enter its hospitals, the victims of its effects on the constitution. It is a question that is asked of every patient, how long he has been in Paris? The first month is the most trying period; before, then, the student commences his labours, he should, to a certain extent, have become habituated to the change of air: his constitution should have time to accustom itself to the peculiarities of difference.

*What Hospitals are worth attending?*—Of the Parisian hospitals there are none that offer such a concentration of medical skill as La Charité. This is situated in the rue des Sts. Pères, and contains between five and six hundred patients. MM. Andral, Cruveilhier, Velpeau, Rayer, Bouillaud, Fouquier, are all to be found within its wards.

He who would learn accurate diagnosis, who would study the use of the stethoscope in its mean, and not in its extreme; he who would learn pathology, by close attendance to the dead house, should follow the service of M. Andral. There are few so free from that national error, excitement, as this celebrated man.

No hospital is better known to us by name than Hôtel Dieu. It was the largest in Paris, containing at one time 1200 beds, but its size is much diminished owing to the improvements which are being made in its immediate vicinity, which have rendered it necessary to pull down one of its largest wings. Of the surgeons attached to this hospital are men whose names are familiar to all in the profession, as MM. Roux and Breschet. The former makes his daily visit, at half-past six in the summer, and at seven in the winter months, and this is followed by his clinical lecture in the amphitheatre of the hospital. M. Breschet commences his rounds at nine o'clock.

There are no less than ten physicians attached to this hospital. Amongst the most celebrated are Chomel, Magendie, and Louis. M. Chomel lectures from nine to ten on Mondays, Tuesdays, and Fridays.

Another hospital that is generally visited by foreigners is the Hôpital St. Louis, in which an opportunity is particularly afforded of studying diseases of the skin. It is situated within five minutes' walk of the rue du faubourg St. Martin. One of the streets leading out of this, called the rue des Recollets, will be found the direct way to this hospital, which is situated in the rue de l'Hôpital, directly in front of the canal that is at the end of the rue des Recollets. Here it was that Alibert and Biett collected their vast mass of information, and were to be found morning after morning, in the midst of their squalid band of patients. But now no more remains of them than a dirty marble bust of the former,—soiled by the hands of the numerous patients that mount the staircase where it is placed—to mark the haunt of this once celebrated man. Time makes sad changes in our profession as in every other, and to this the Hôpital St. Louis bears ample testimony. MM. Lugol and Emery are the only remains of the former days of St. Louis.

M. Lugol is, in every sense of the term, a great man. He styles himself "Le grand lustre du monde." It is curious to follow him in his scrofulous wards, and there hear him descant on the miraculous powers of iodine.

Near to the Hôpital des Veneriens is the Maison d'Accouchement, or Hospice de la Maternité. There is much difficulty in gaining admission here, as no medical man has a right within its wards, excepting those that are officially attached to it. There are between four and five hundred beds within it.

The Hospice de la Salpêtrière is an establishment on a very large scale. It contains between five and six thousand beds, entirely for women. Of these a certain number are appropriated to old and infirm persons, others are set apart for patients labouring under incurable diseases. Here it was that M. Cruveilhier compiled his admirable work on pathological anatomy, the dead-house affording no small supply of diseases, in their most hideous forms. A certain portion of this establishment is allotted to those who are of unsound mind, their number varying from a thousand to twelve hundred.

The Bicêtre is for men what the Salpêtrière is for women.

*Parisian Dissecting Rooms versus the English.*—True it is that subjects for dissection are far more numerous than those in England. In this point there is certainly a superiority. When, however, the accommodations that are provided in the Parisian dissecting rooms are taken into consideration, the preference will be given to those of our own country. Let the student who has been accustomed to the dissecting rooms in England enter those in Paris: the stench which first assails his nasal organs is almost insupportable, from the system of smoking which is carried on within them. Again, there is nothing like decency or order kept up; portions of viscera, detached limbs, pieces of dissected muscle, fat, and cellular membrane, are seen to cover the floor. It is necessary to be careful how we tread, lest we should stumble against some limb lying in our way, or slip up, from stepping on some viscid substance that may be strewed upon the ground.

There are two great dissecting schools in Paris. One is close to the Ecole de Médecine, and adjoins Dupuytren's museum of morbid anatomy; the other is situated near the hospital of La Pitié, and is called Clamart. This is one of a more modern date than the former, to which it is thought preferable. It is certainly situated in a more airy neighbourhood, and its accommodations are somewhat of a better order. It is necessary for all those who intend to dissect, to become pupils of the Internes, who have the choice of all subjects brought for dissection. Each interne has four pupils, who are attached to one subject, that is usually changed every ten days or a fortnight. The sum for the season, which lasts about five months, is about 150 francs, or six pounds. To those who would be free from the inconveniences of the common dissecting rooms, a means is offered of dissecting in private, by becoming the pupils of the overseer of the school, who has private rooms set apart for this purpose. He of course demands a sum of money in proportion to the convenience offered, and this more especially of the English, who are always supposed to have a superabundance of money, wherewith they can afford to pay handsomely.

